# Impaired Reproductive Function in Equines: From Genetics to Genomics

**DOI:** 10.3390/ani11020393

**Published:** 2021-02-03

**Authors:** Nora Laseca, Gabriel Anaya, Zahira Peña, Yamila Pirosanto, Antonio Molina, Sebastián Demyda Peyrás

**Affiliations:** 1 Departamento de genética, Universidad de Córdoba, Campus de Rabanales Ctra, Madrid-Cádiz, km 396, 14071 Córdoba, Spain; noralaseca@gmail.com (N.L.); ganaya@gmail.com (G.A.); zahiramaria@hotmail.com (Z.P.); ge1moala@uco.es (A.M.); 2 Departamento de Producción Animal, Facultad de Ciencias Veterinarias, Universidad Nacional de La Plata, La Plata 1900, Argentina; yamipiro@gmail.com; 3 Consejo Superior de Investigaciones Científicas y Tecnológicas (CONICET), CCT-La Plata, La Plata 1900, Argentina

**Keywords:** reproductive traits, fertility, mares, stallions, genomics, candidate genes, copy number variations, inbreeding, genome-wide association studies

## Abstract

**Simple Summary:**

The genetic origins behind reproductive traits are still far from clear: this is especially true in horses, where the lack of objective reproductive phenotypes (particularly in mares) reduces the quantity of information available. However, in recent years, the study of genomics has produced a notable increase in our knowledge of genetic causes of reproductive impairment in the species. In this paper, we review the recent advances and studies analyzing genomic mechanisms affecting the reproductive function in mares and stallions.

**Abstract:**

Fertility is one of the key factors in the economic and productive success of the equine industry. Despite this, studies on the genetic causes affecting reproductive performance are scarce, especially in mares, where the genetic architecture of the reproductive traits is extremely complex. Today, with the increasing availability of new genomic methodologies for this species, we are presented with an interesting opportunity to understand the genetic basis of equine reproductive disorders. These include, among others, novel techniques for detecting chromosomal abnormalities, whose association with infertility in horses was established over 50 years ago; new sequencing technologies permitting an accurate detection of point mutations influencing fertility, as well as the study of inbreeding and molecular homozygosity, which has been widely suggested as one of the main causes of low reproductive performance in horses. Finally, over the last few years, reproductive performance has also been associated with copy number variants and candidate genes detected by genome-wide association studies on fertility traits. However, such studies are still scarce, probably because they depend on the existence of large and accurate phenotypic datasets of reproductive and/or fertility traits, which are still difficult to obtain in equines.

## 1. Introduction

Reproductive traits are genetically heterogeneous and complex, as they are usually determined by the allelic combinations of multiple genes. They also have low heritability [1], which makes them particularly sensitive to environmental and management factors (e.g., age, nutrition, training, temperature at mating, and breeding season, among others) By that reason, modeling reproductive traits from a genetic point of view is difficult. This is particularly important in equines, whose fertility is considerably lower than that observed in other domestic species. In addition, equine reproductive efficiency is limited by their own physiology, which is characterized by single births in almost all the foalings, seasonality in mares, long generation intervals delaying genetic improvement, and a lack of systematized collection of phenotypic information reproductive traits. Nevertheless, a certain degree of genetic influence has been described and modelized from a quantitative viewpoint, in mares and stallions. For instance, gestation length in mares is affected by maternal lineage or inbreeding [2,3,4]. We also recently reported the influence of the breed and individual in six fertility traits in mares belonging to 8 different Spanish breeds [5]. Similarly, changes in sperm traits were related to the genetic background of the individual, lineage and breed [6,7,8], as well as by the inbreeding value [9,10]. However, there are very few reports which describe the molecular mechanisms involved in such genetic influence or which detect the candidate genes involved in the biological processes.

Today, the advent of genomics has led to the development of new methodologies for genetic analysis in livestock animals. These techniques, which are largely employed in humans and laboratory models, include the detection of specific mutations and/or deletions (Indels), copy number structural variants (CNVs) [11], runs of molecular inbreeding (ROHs) [12], and genome-wide association studies (GWAS) [13]. However, their use in equines is still limited, probably due to the delay in the development of a reliable reference genome, in comparison with most livestock species, such as pigs or cattle, but also by a lack of reliable expected progeny differences (EPDs) and phenotypic values associated to the variations in fertility in the species. Moreover, although the use of genomic methods in horses has increased significantly over the past five years [14], studies focusing on reproductive traits are still few and far between. Even less are those which aim to dissect and quantify more accurately the influence of the genetic background and the environment in the expression of the phenotype. Here, we aim to review the recent advances in our understanding of some of the genomic mechanisms involved in impaired reproductive function in horses.

## 2. Mutations, Deletions and Genomic Rearrangements Associated with Infertility in Horses

Deletions were first associated with reduced fertility in horses in 1995, when Pailhoux, et al. [15] detected a deletion in the sex-determining region Y (SRY) which was responsible for infertility in mares carrying a 64,XY chromosomal complement. This gene, located in the Y chromosome, was later related to the initial development of the testis (rather than ovaries) in the early pluripotential gonad, by upregulating *SOX9* and *SF1* and increasing the production of anti-Müllerian hormone (AMH) (Table 1) [16]. More recently, Raudsepp, et al. [17] determined that SRY deletion is frequent, accounting for one of four individuals with chromosomal abnormalities. This syndrome, which is not detected in other species, is associated in most cases with the infertile mare phenotype characterized by different extents of ovarian and uterine dysgenesis [17,18,19], and it could also be related to the distinctive organization of the Y chromosome in the horse (a single copy located in the proximal region of the q arm of horse Y chromosome(ECAY)), which makes it more prone to deletion.

In stallions, the identification of mutations related to fertility problems is also scarce. Révay, et al. [24] identified a start codon mutation (c.1A > G) in the androgen receptor (AR) gene which was linked to androgen insensitivity syndrome (AIS) (Table 1). Several years later, a new familial androgen receptor mutation in horses was reported by Bolzon, et al. [25], where a missense mutation (c.2042G > C) at AR exon 4 explained the segregation of the disorders of sexual development (DSD) in a Thoroughbred horse family. This mutation, which was expected to affect the ligand-binding domain of the AR protein, led to complete androgen insensitivity of 64,XY SRY+ testicular DSD individuals. In 2017, the same authors reported a 25-bp hemizygous deletion including 8 codons in exon 2 (c.1630_1654del) in 4 Warmblood mares with sex reversal genotype (64, XY) and equine testicular feminization syndrome (AIS). More recently, two novel variants were detected in the AR gene in horses, including a novel deletion in exon 1 and point mutation on exon 5 [21]. These serial studies, performed over more than 8 years, demonstrated that mutations and deletions in the AR gene are causative of equine AIS and can, therefore, be associated with discordances between chromosomal and phenotypic sex in this species. 

Autosomal mutations and rearrangements have also been related to infertility in horses: Ghosh, et al. [20] reported, in the first screening of copy number variations (CNVs) performed in the species, a deletion on *Equus caballus* chromosome ECA29 including *AKR1C* gene. More recently, Ghosh, et al. [23] screened the same mutation in 622 horses with reproductive or sex developmental problems and revealed an increased frequency (8–9%) compared to fertile horses used as the control (Table 1). Besides, 4 out of every 5 individuals carrying a homozygous deletion were reproductively abnormal, with a particular increase in the incidence of cryptorchidism. Similar deletions on *AKR1C* genes, which are actively involved in the biosynthesis of androgens and estrogens, have previously been linked to sexual development dysgenesis in humans [26]. Here, the authors suggested that such deletions could be considered as a risk factor for equine reproductive disorders. On the other hand, autosomal translocations have also been related to a reduction in fertility in several cases [27,28,29]. Moreover, a de-novo balanced translocation t(12q;25) was recently detected in a cloned Arab horse [23]. Even though all the cases showed no loss of genetic material or genes affected, the individuals presented diminished fertility due to repeated early embryonic losses (REELs). 

## 3. Copy Number Abnormalities and Fertility: the Role of the Sex Chromosome Pair

Genomic abnormalities characterized by a variation in the number of copies of a particular sequence from one individual in comparison with the reference genome of the species are known as copy number alterations [30]. The most common of these are changes in the chromosomal number of an individual compared with the standard karyotype of the species, known as copy number aberrations (CNA). In horses, CNAs are particularly relevant, as shown by the greater number of cases reported than in the rest of domestic animals [31]. CNAs were first related to infertility in horses nearly 50 years ago [32,33]. Fifteen years later, Power [34] reported up to 400 individuals carrying CNAs, of which over 95% were related to fertility problems. Thirty years later, it became clear that ECAX monosomy, in its true (63,X; [35]) or 64,XX/63,X mosaic [36] forms, together with 64,XYdsd sex reversal mares [19], are the most common presentations in the species. Both cases are associated with a normal mare phenotype with a lack of development of the internal reproductive organs [37]. However, two more syndromes were also widely reported: 64, XXdsd (sex reversal or pseudohermaphrodite males) and 64,XX/64,XY (sex chimerism). The former cases are usually associated with ambiguous genitalia, fused vulva and an enlarged clitoris, and are, therefore, easily detected [37,38]. Conversely, sex pair chimerism was associated with normal [39] and abnormal [40] phenotypes, which results in a much lower detection rate. Finally, some abnormal complex karyotypes, including sex-pair [41] and autosomal [28] chromosomes, were also related to lack of fertility in the species. This increased prevalence observed, in comparison with other livestock species, is quite remarkable and mainly due to the complexity of the horse karyotype and the lack of availability of equine cytogenetic laboratories in several countries [42]. However, new genomic technology, based on short tandem repeats (STRs) [43], droplet digital PCR (ddPCR) [44], and, more recently, single nucleotide polymorphism (SNP) genotyping array [45], is being constantly developed. There is, therefore, likely to be an increase in the number and complexity of chromosomal syndromes associated with infertility detected in horses in the near future.

## 4. Copy Number Variants: A New Field for Horse Genomics and Fertility

Several years ago, the existence of submicroscopic changes (including deletions, insertions, duplications, and complex multi-site variants) of DNA segments across the genome of all the individuals was described [46]. These variations are characterized by reduced size, ranging from kilobases (kb) to megabases (Mb), which can, therefore, affect the number of copies of a particular gene (or region) without affecting an individual’s karyotype. However, small changes of this kind cannot be detected by classic or molecular cytogenetic techniques. In humans, copy number variations constitute a structural polymorphism which has great functional relevance as it is an important source of phenotypic and genotypic variation. They have also been associated with diseases and failures in sexual development and reproduction [47]. In horses, studies describing CNVs at the genome-wide level are relatively novel and scarce, especially those related to fertility. It was in 2012 when the first report of copy number variation in horses was published, which suggested that CNVs are common in the horse genome and may modulate the biological processes underlying different traits [48]. In particular, the authors detected a CNV gain (duplication) overlapping the bone morphogenetic protein receptor-1B gene (*BMPR1B*), which had previously been associated with fecundity in small ruminants. However, it was only proposed as a candidate gene involved in the regulation of ovulation rate, since the small number of samples evaluated did not allow them to make a conclusive association. 

Two years later, Ghosh et al. [20] analyzed CNVs in 40 individuals of 16 breeds, including two Przewalski horses, reporting CNV regions overlapping with several genes associated with the reproductive system. Among these, *BMPR1B* and zona pellucida binding protein (*ZPBP*), associated with oocyte quality (Table 2) and sperm-oocyte interaction, respectively, were affected (Table 3). The same study revealed CNV regions overlapping several genes involved in the steroid metabolism, including *HSD17B6*, *CBRr*, *PHYH*, and *UCMA*, among others, suggesting that they could be partially involved in variations in the sexual development of the individuals (Table 1). Finally, the same study determined that CNV losses also overlapped with several well-known genes involved in different processes of sperm biology, such as spermatogenesis (*IFT81*, *ZNF331*) (Table 4), sperm capacitation and binding to the oocyte (*ELSPBP1*, *SP-1*, *BSP2*, *BSPH1*, *SULT2A1*) (Table 3), and maturation and fertilization capacity (*L1TD1*, *ADAM20*) (Table 4). Finally, the largest study assessing CNVs in horses (1755 individuals belonging to 8 European breeds) was recently published by Sole, et al. [49]. The authors identified several regions of CNVRs which overlapped candidate genes previously associated with steroidogenesis (*LCN6*), spermatogenesis (*FKBP6* and *SOHLH1*), sperm movement (*DNAH7*) (Table 4), sperm-oocyte binding ability (*MFGE8) (*Table 3), and stallion fertility [50,51,52,53], suggesting that CNVs could be a source of fertility variation among individuals. However, none of the candidate genes detected by this methodology were further validated by specific studies; therefore, the association between copy number variants and fertility still needs to be analyzed in greater depth.

Overall, horses seem to be particularly affected by structural changes across the genome. However, large scales studies associating fertility and genomic structural variations are still scarce in comparison with other domestic animals, such as pigs or cattle [22,62]. We, therefore, believe that the association between specific copy number variants and reproduction is an interesting field to explore, in which there are more genetic mechanisms associated with fertility traits in horses to be unveiled.

## 5. Inbreeding, Molecular Homozygosity and Reproduction in Horses

Inbreeding is the reduction in genetic variability in a particular individual or population driven by the mating of related individuals. Their phenotypic effect is a decrease in biological fitness, known as inbreeding depression. From a genetic point of view, inbreeding increases the homozygosity of the individuals (and populations) triggering the phenotypic expression of recessive deleterious mutations [63]. By this reason, it has also been pointed out as one of the most important causes of reduced fertility in wildlife [64], as well as livestock populations [65,66]. In horses, inbreeding rates are normally higher in comparison with other livestock species for several reasons, such as the existence of breeds with very small effective population sizes [67,68] or the development of breeding schemes focused mainly on morphological traits [69] or athletic performance [70,71], or the lack of genetic control of mating [72].

During the last 10 years, different approaches and methodologies have been developed to analyze the effect of inbreeding at the genomic level. Of these, the most important approach employed has been the detection of large homozygous genomic regions known as runs of homozygosity (ROH). The use of these techniques, originally developed for studying the genomic basis of diseases in humans [73], has rapidly spread to livestock animals due to the increased accuracy and reliability of the inbreeding estimations provided [74,75]. Besides, this methodology has allowed us to determine genomic regions at the population level, as well as metabolic pathways associated with individuals sharing common phenotypes, and is viable even in traits with unknown, complex genetic architectures, such as those related to reproduction and fertility [76]. However, despite the proven link between inbreeding and reproduction in livestock species, the ROH studies focused on fertility traits were performed in horses.

To our knowledge, the first report assessing molecular homozygosity and reproductive parameters in stallions was published in 2003, although no significant correlations were found [77]. However, it was performed using information from 11 STR markers, thus reducing the possibility of finding significant associations. Twelve years later, Metzger, et al. [55] reported the first study based on the detection of ROH regions in six selectively-bred and non-bred horse breeds, in which a significant increase of inbreeding was detected in the genomic regions comprising 139 genes. Among these, two regions located on ECA22 and ECA8 included *SPATA25* and *YES1* genes (Table 4), which were associated with obstructive azoospermia and self-defensive mechanisms in spermatocytes, respectively. Similarly, a region located on ECA3 included *FRAS1* (Table 3), which had previously been associated with impaired embryonic development of internal organs in mice. Another ROH-enriched region located on ECA28 (14,656,676–14,778,472) was also found to include the *KIT* ligand gene (*KITLG*) (Table 3), associated with gametogenesis and embryonic development in humans and mice. This gene has been associated with the dominant white coat color locus (W) which produces lethal disorders in the very early stages of gestation (W/W-genotype) [58], thus affecting fertility by indirect pathways. Similarly, recently, Velie, et al. [78] identified a negative effect of inbreeding on ECA1 quantitative trait loci (QTL) in a large population of Norwegian-Swedish Coldblooded trotters. However, the same region was previously reported by Gottschalk et al. [50] in association with the number of motile sperm, suggesting that the reduction in fertility from inbreeding could be partially mediated by a reduction in sperm quality (Table 4). This is in agreement with our preliminary results, in which we detected a reduction in total and progressive motility in 94 Pura Raza Español (PRE) stallions triggered by an increase of inbreeding levels (F_ROH_) beyond a certain threshold [9]. In addition, we recently reported the first negative association between SNP-based inbreeding values (F_ROH_) and the foaling number expected breeding value (EBV_FN_) in 243 PRE mares [79]. However, our results also showed that the increase in ECAX homozygosity (F_ROHX_) was even more closely associated with a reduction in the EBV_FN_ than the homozygosity observed in the rest of the genome. All these studies point to the existence of specific pathways in which the decreased intra-loci variability of specific genes could be a partial cause of decreased fertility in horses. However, further validations are still needed. 

Using a similar approach, two recent studies have also given an interesting overview of an indirect pathway by which inbreeding could affect fertility in horses. In 2019, Orlando and Librado [80] reported an increased load of deleterious mutations in horses with increased inbreeding levels. Those mutations were associated with cerebellar abiotrophy (ECA2), hydrocephalus (ECA1), and congenital liver fibrosis (ECA20), among other disorders. Similarly, Todd, et al. [57] identified a lethal embryonic haplotype in the *LY49B* gene on ECA6 which also showed high frequencies of heterozygotes in thoroughbreds, Norwegian-Swedish Coldblooded Trotters and Swedish Warmbloods (Table 3). Both studies indicate, from a genomic point of view, the importance of the partial dominance hypothesis [81], in which an increase in the expression of deleterious recessive alleles due to increased inbreeding could trigger a reduction in fertility by increasing embryonic lethality. These findings also are supported by the fact that the usual breeding practices employed in horses do not take into account the fertility of the individuals, thus favoring the permanency of deleterious genes and reducing the inbreeding purge over time [82]. 

Finally, two recent studies were recently published using a F_ROH_-based approach in combination with the detection of selection sweeps detecting candidate genes related to fertility. In the first of these, Ablondi, et al. [56] analyzed the outcome of selective pressures acting on 382 horses bred for different purposes in a short evolutionary time. The authors detected distinctive genomic footprints among the groups, some of which were related to genes with a proven reproductive effect, such as the *ZPBP* gene on ECA4 (Table 3), which has recently been related to sexual development in cattle [83]. Similarly, Gurgul, et al. [54] detected several genes involved in the fertility of mares which were differentially affected among breeds, including processes related to oocyte maturation (*PRKACA*, *ANAPC5*, *ANAPC7*), oocyte meiosis (*ANAPC5*, *ANAPC7*, *ADCY1*) and ovarian steroidogenesis (*ADCY1*) (Table 2). Despite the fact that the results obtained in both cases were not validated, the detection of candidate genes related to fertility traits showing differences among populations, together with combined approaches, could be an interesting alternative to explore in the future. 

Overall, the existing reports strongly suggest the existence of a negative effect produced by increased homozygosity in certain regions of the genome on reproductive traits in mares and stallions. However, the information available is still scarce, particularly in mares, in comparison with other livestock populations. Therefore, genomic-based inbreeding studies are undoubtedly an interesting field to explore further to achieve a better understanding of the genomic architecture of horse fertility traits. 

## 6. Association Studies and Fertility in Horses

Genomic association studies are a fundamental tool to detect the genes and mechanisms involved in the regulation of a specific trait or biological process [84]. They aim to associate a certain allelic combination of a single locus or group of loci with a percentage of the variation observed in the respective phenotypes. However, they are based on previous findings to determine which gene or region should be tested against a particular phenotype. In contrast, genome-wide association studies (GWAS) [13] are based upon the principle of non-random association between alleles at different loci (linkage disequilibrium) and a particular phenotype at the population level and, therefore, could be used to scan the entire genome in the search of causative variants. This technique has been successfully employed to detect candidate genes associated with fertility traits in several livestock species, such as cattle [85] or sheep [86]. However, their use in horses has been delayed, probably until the development of high-density SNP genotyping arrays based on accurate reference genomes, which could provide a certain degree of reliability in the candidate genes detected.

In stallions, one of the first associations between a candidate and fertility (*CRISP3)* (Table 4) was described nearly 20 years ago [60], and was further validated with proven certainty by the same group five years later [61]. Thereafter, more than 60 candidate genes have been described in horses, mostly related to stallion fertility, including *SPATA1*, *INHBA*, *ACE*, *SP17*, *FSHB*, *PRLR* [87,88,89,90], *PLCz1* [51], and *FKBP6* [52,59] (Table 5). 

However, it was not until 5 years ago that Schrimpf et al. [53] performed the only genome-wide scanning study in horses, in which several candidate genes associated with fertility traits in stallions were reported (Table 5). Among these, the most important finding was an association between an SNP and *NOTCH1* (g.37,453,246G > C) which produced a significant effect mediated by disruption on a splicing site. However, the same study reported 7 additional loci with harboring variants with a deleterious effect on stallion fertility, including *CFTR*, *OVGP1*, *FBXO43*, *TSSK6*, *PKD1*, *FOXP1*, and *TCP11* genes. Nevertheless, all these results should be taken with caution, since the analysis was only performed in Hannoverian horses; therefore, their validation on other breeds or equine populations is still pendant.

In mares, de Leon, et al. [92], reported an association between variants on the *P53* gene and abortion after analyzing 105 Thoroughbred individuals, demonstrating the existence of candidate genes with a proven relationship with reproductive efficiency in mares (Table 5). More recently, El-Sheikh Ali, et al. [91] reported 12 genes differentially expressed in mares with placentitis (Table 5). Even though both cases were focused on reproductive diseases, and the latter was performed using a transcriptomic approach, those results suggest that differences in non-infectious abortions could be partially mediated by genetic mutations. Meanwhile, GWAS studies performed on reproductive traits are still lacking in mares. While there could be several causes for this lack of experimental results, it is highly likely that the difficulty in obtaining large-scale, accurate phenotypic datasets and the difficulty of modeling the environmental effects on such traits in the species are the most important [5].

Overall, the number of single association or GWAS studies focused on horse reproduction is still extremely small in comparison with other livestock species. This situation gives us an interesting opportunity to determine more accurately the genetic basis of horse reproduction, which is of great importance in a species which is well-known for reduced fertility. However, we believe that the lack of phenotype traits is still a major problem to be solved before large-scale dissemination of association studies in horses is possible.

## 7. Conclusions and New Approaches for Studying the Genes Involved in Equine Reproductive Problems

Horse genomics is currently undergoing an exponential expansion, not least due to the adaptation of new genomic methodologies to the species, the existence of a new, accurate reference genome [93], and the exponential increase in the number of equines which have been genotyped [94]. It is, therefore, highly likely that our knowledge of the reproductive architecture of horses will grow considerably over the next few years. However, large-scale datasets of reproductive phenotypes are still scarce in horses, probably due to the lack of availability of reliable reproductive phenotypes (particularly in mares). Therefore, the development of new phenotypes to measure reproductive fitness more objectively and their systematic use by breeder associations are essential to allow a more in-depth study of the reproductive function in horses.

In addition, the search for candidate genes is a highly promising methodology to obtain a better understanding of the processes involved in horse fertility. They not onlycan help to elucidate which physiological functions could be affected by a specific genotype, but also to predict which genotypes could be more affected by environmental challenges, and they can be integrated into breeding programs to detect, even at very young ages, the potential fertility (increased, normal, or decreased) of a given mare or stallion. In addition, the lack of consistency observed in the candidate genes associated with fertility detected in different breeds or populations of horses, (most of them detected by a single study; see Table 1, Table 2, Table 3, Table 4 and Table 5) will be only reduced with an increase in the volume of evidence gathered. In this sense, only 11 genes reviewed in this manuscript were detected by two or more studies, and only 5 were detected using more than one approach. It has recently been demonstrated that combined genomic approaches in the same study can increase accuracy and reliability in detecting candidate genes [95,96]. However, no combined studies have yet been reported in horses. Such combined approaches would constitute the best approach in our search for a better, more reliable understanding of genetic effects on horse fertility.

## Figures and Tables

**Table 1:** Candidate genes related to problems in gonadal or sexual development in equines.

Gene	Name	Position	Approach	Reference
*HSD17B6*	Hydroxysteroid 17-beta dehydrogenase 6	ECA7: 3,935,674-3,938,482	CNV	[20]
*SOX9*	SRY-Box Transcription Factor 9	ECA11: 9,224,053-9,229,840	Mutations/Deletions	[16]
*SF1*	Splicing factor 1	ECA12: 28,619,898- 28,632,463	Mutations/Deletions	[16]
*AR*	Androgen receptor	ECA12: 26,039,218-26,041,649	Mutations/Deletions	[21,22]
*PHYH*	Phytanoyl-CoA 2-Hydroxylase	ECA29: 22,540,934- 22,563,145	CNV	[20]
*UCMA*	Upper zone of growth plate and cartilage matrix associated	29: 22,681,823- 22,691,596	CNV	[20]
*AKR1C*	Aldo-keto reductase family 1 member C	ECA29: 29,700,000- 29,900,000	CNV	[20,23]
*CBRr*	Campylobacter bile resistance regulator	ECA29: 32,837,886- 32,838,194	CNV	[20]
*SRY*	Sex determining region	ECAY	Mutations/Deletions	[15,17,18]

ECA: *Equus caballus* chromosome; CNV: copy number variants.

**Table 2:** Genes related to oocyte development in equines.

Gene	Name	Position	Approach	Reference
*BMPR1B*	Bone morphogenetic protein receptor-1B	ECA3: 44,402,722- 44,692,141	CNV	[20,48]
*ADCY1*	Adenylate cyclase 1	ECA4: 16,027,025- 16,171,067	ROH	[54]
*PRKACA*	Protein kinase cAMP-activated catalytic subunit alpha	ECA7: 46,048,251- 46,065,141	ROH	[54]
*ANAPC5*	Anaphase promoting complex subunit 5	ECA8: 24,310,740- 24,348,719	ROH	[54]
*ANAPC7*	Anaphase promoting complex subunit 7	ECA8: 23,907,492- 23,927,512	ROH	[54]
*LRRC6*	Leucine rich repeat containing 6	ECA9: 75,402,662- 75,588,283	Candidate gene	[50]
*ATP6V1E2*	ATPase H+ transporting V1 subunit E2	ECA15: 53,416,247- 53,416,927	Candidate gene	[50]

CNV: Copy number variation, ROH: runs of homozygosity.

**Table 3:** Genes related to gametic interaction and embryo development in equines.

Gene	Name	Position	Approach	Reference
*MFGE8*	Milk fat globule *EGF* and factor V/VIII domain containing	ECA1:95,221,735- 95,253,405	CNV	[49]
			Candidate gene	[50]
*FRAS1*	Fraser extracellular matrix complex subunit 1	ECA3: 59,404,529- 59,818,746	ROH	[55]
*ZPBP*	Zona pellucida binding protein	ECA4: 19,776,870-19,907,352	CNV	[20,49]
			ROH	[56]
*LY49B*	Killer cell lectin-like receptor	ECA 6: 39,335,921- 39,347,553	ROH	[57]
*UBBP4*	Ubiquitin B pseudogene 4	ECA8: 24,467,333- 24,468,548	CNV	[49]
*SP-1*	*Sp1* transcription factor	ECA10: 14,480,982- 14,485,022	CNV	[20]
*BSP2*	Binder of sperm 2	ECA10: 14,481,079-14,506,004	CNV	[20]
*SULT2A1*	Sulfotransferase family, cytosolic, 2A, dehydroepiandrosterone (DHEA)-preferring, member 1	ECA10: 18,124,115- 18,322,483	CNV	[20]
*BSPH1*	Binder of sperm protein homolog 1	ECA10: 18,375,988- 18,377,065	CNV	[20]
*ELSPBP1*	Epididymal Sperm Binding Protein 1	ECA10: 18,397,898- 18,416,427	CNV	[20]
*PLOD3*	Procollagen-lysine,2-oxoglutarate 5-dioxygenase 3	ECA13: 9,454,913- 9,462,562	CNV	[49]
*KITLG*	*KIT* ligand	ECA28: 15,726,503- 15,807,871	ROH	[55]
			Candidate gene	[58]

CNV: Copy number variation, ROH: runs of homozygosity.

**Table 4:** Genes related to sperm quality traits in equines.

Gene	Name	Position	Approach	Reference
*HERC4*	*HECT* and *RLD* domain containing E3 ubiquitin protein ligase 4	ECA1: 56,815,617- 56,954,039	Candidate gene	[50]
*MFGE8*	Milk fat globule EGF and factor V/VIII domain containing	ECA1:95,221,735- 95,253,405	CNV	[49]
			Candidate gene	[50]
*SPATA48*	Spermatogenesis associated 48	ECA4: 19,909,625- 19,963,732	ROH	[56]
*MIER1*	*MIER1* transcriptional regulator	ECA5: 91,061,840- 91,119,716	Candidate gene	[50]
*L1TD1*	*LINE1* type transposase domain containing	ECA5: 95,020,437- 95,025,347	CNV	[20]
*IFT81*	Intraflagellar transport protein 81 homolog	ECA8: 24,053,812- 24,132,668	CNV	[20]
*YES1*	*YES* proto-oncogene 1, Src family tyrosine kinase	ECA8: 44,273,501- 44,304,857	ROH	[55]
*FKBP6*	*FKBP* prolyl isomerase 6	ECA13: 11,350,401- 11,378,073	CNV	[49]
			Candidate gene	[52,59]
*DNAH7*	Dynein axonemal heavy chain 7	ECA18: 71,435,145- 71,669,919	CNV	[49]
*ZNF331*	Zinc finger protein 331	ECA20: 28,318,795- 28,329,094	CNV	[20]
*CRISP3*	Cysteine-rich secretory protein 2	ECA20: 48,708,574- 48,761,076	Candidate gene	[50,60,61]
*CRISP1*	Cysteine rich secretory protein 1	ECA20: 48,856,838- 48,887,485	Candidate gene	[50]
*SPATA25*	Spermatogenesis associated 25	ECA22: 35,747,531- 35,748,590	ROH	[55]
*ADAM20*	*ADAM* metallopeptidase domain 20	ECA24: 16,539,958- 16,547,675	CNV	[20]
*SOHLH1*	Spermatogenesis and oogenesis specific basic helix-loop-helix 1	ECA25: 38,791,446- 38,797,028	CNV	[49]
*GLIPR1L1*	*GLIPR1* like 1	ECA28: 4,284,550-4,323,990	Candidate gene	[50]

CNV: Copy number variation; ROH: runs of homozygosity.

**Table 5:** Genes related to fertility trait in equines.

Gene	Name	Position	Approach	Reference
*GJA4*	Gap junction protein alpha 4	ECA2: 22,443,340-22,444,341	Candidate gene	[91]
*CXCL2*	Chemokine (C-X-C motif) ligand 1 (melanoma growth stimulating activity, alpha)	ECA3: 63,470,014-63,586,176	Candidate gene	[91]
*INHBA*	Inhibin subunit beta A	ECA4: 12,760,757-12,808,658	Candidate gene	[90]
*CFTR*	CF transmembrane conductance regulator	ECA4: 74,741,421-74,918,780	Candidate gene	[53]
*PTGS2*	Prostaglandin-endoperoxide synthase 2	ECA5: 20,490,127-20,497,264	Candidate gene	[91]
*S100A8*	S100 calcium binding protein A8	ECA5: 40,744,248-40,744,667	Candidate gene	[91]
*S100A9*	S100 calcium binding protein A9	ECA5: 40,778,743-40,821,668	Candidate gene	[91]
*OVGP1*	Oviductal glycoprotein 1	ECA5:53,508,181- 53,522,618	Candidate gene	[53]
*SPATA1*	Spermatogenesis associated 1	ECA5: 76,122,099-76,165,463	Candidate gene	[87]
*PTGER3*	Prostaglandin E receptor 3	ECA5: 87,780,622-87,951,028	Candidate gene	[91]
*PLCz1*	Phospholipase C zeta 1	ECA6: 46,812,109-46,852,694	Candidate gene	[51]
*RETN*	Resistin	ECA7: 5,460,957-5,462,512	Candidate gene	[91]
*MMP1*	Matrix metallopeptidase 1	ECA7: 13,098,650-13,176,364	Candidate gene	[91]
*SP17*	Sperm autoantigenic protein 17	ECA7: 34,254,555-34,264,346	Candidate gene	[88]
*RLN*	Relaxin 3 RLN 3	ECA7: 46,105,165-46,106,720	Candidate gene	[91]
*FSHB*	Follicle stimulating hormone beta subunit	ECA7: 98,422,248-98,424,267	Candidate gene	[88]
*FBXO43*	F-box protein 43	ECA9:45,973,733-45,985,463	Candidate gene	[53]
*ACE*	Angiotensin I converting enzyme	ECA11: 15,802,359-15,822,526	Candidate gene	[88]
*FKBP6*	FKBP prolyl isomerase 6	ECA13: 11,350,401-11,378,073	Candidate gene	[52,59]
*PKD1*	Polycystin 1, transient receptor potential channel interacting	ECA13: 41,880,905-41,926,116	Candidate gene	[53]
*FOXP1*	Forkhead box P1	ECA16: 20,353,146-20,717,328	Candidate gene	[53]
*TCP11*	T-complex 11	ECA20: 36,147,583-36,279,044	Candidate gene	[53]
*TSSK6*	Testis specific serine kinase	ECA21: 4,554,495-4,555,316	Candidate gene	[53]
*PRLR*	Prolactin receptor	ECA21: 31,054,801-31,107,331	Candidate gene	[89]
*P53*	P53 and DNA damage regulated 1	ECA22: 23,560,441-23,566,500	Candidate gene	[92]
*PI3*	Peptidase inhibitor 3	ECA22: 35,155,086-35,157,165	Candidate gene	[91]
*NOTCH1*	Notch receptor 1	ECA25: 38,056,617-38,104,337	Candidate gene	[53]
*APOBEC3Z1B*	Apolipoprotein B mRNA-editing enzyme-catalytic polypeptide-like 3Z1b	ECA28: 37,062,159-37,065,847	Candidate gene	[91]

## Data Availability

Data reviewed in this study was obtained from public databases.
